# Afatinib and Temozolomide combination inhibits tumorigenesis by targeting EGFRvIII-cMet signaling in glioblastoma cells

**DOI:** 10.1186/s13046-019-1264-2

**Published:** 2019-06-18

**Authors:** Raghupathy Vengoji, Muzafar A. Macha, Rama Krishna Nimmakayala, Satyanarayana Rachagani, Jawed A. Siddiqui, Kavita Mallya, Santhi Gorantla, Maneesh Jain, Moorthy P. Ponnusamy, Surinder K. Batra, Nicole Shonka

**Affiliations:** 1 Department of Biochemistry and Molecular Biology, University of Nebraska Medical Center, Omaha, NE 68198 USA; 2 Department of Otolaryngology/Head and Neck Surgery, University of Nebraska Medical Center, Omaha, NE 68198 USA; 3 Department of Pharmacology & Experimental Neuroscience, University of Nebraska Medical Center, Omaha, NE 68198 USA; 4 Fred and Pamela Buffett Cancer Center, University of Nebraska Medical Center, Omaha, NE 68198 USA; 5 Eppley Institute for Research in Cancer and Allied Disease, University of Nebraska Medical Center, Omaha, NE 68198 USA; 6 Department of Internal Medicine, Division of Oncology and Hematology, University of Nebraska Medical Center, Omaha, NE 68198 USA

**Keywords:** Temozolomide, Afatinib, Glioblastoma, Cancer stem cells, Epidermal growth factor receptor

## Abstract

**Background:**

Glioblastoma (GBM) is an aggressive brain tumor with universal recurrence and poor prognosis. The recurrence is largely driven by chemoradiation resistant cancer stem cells (CSCs). Epidermal growth factor receptor (EGFR) and its mutant EGFRvIII are amplified in ~ 60% and ~ 30% of GBM patients, respectively; however, therapies targeting EGFR have failed to improve disease outcome. EGFRvIII-mediated cross-activation of tyrosine kinase receptor, cMET, regulates GBM CSC maintenance and promote tumor recurrence. Here, we evaluated the efficacy of pan-EGFR inhibitor afatinib and Temozolomide (TMZ) combination on GBM in vitro and in vivo.

**Methods:**

We analyzed the effect of afatinib and temozolomide (TMZ) combination on GBM cells U87MG and U251 engineered to express wild type (WT) EGFR, EGFRvIII or EGFRvIII dead kinase, CSCs isolated from U87 and U87EGFRvIII in vitro*.* The therapeutic utility of the drug combination was investigated on tumor growth and progression using intracranially injected U87EGFRvIII GBM xenografts.

**Results:**

Afatinib and TMZ combination synergistically inhibited the proliferation, clonogenic survival, motility, invasion and induced senescence of GBM cells compared to monotherapy. Mechanistically, afatinib decreased U87EGFRvIII GBM cell proliferation and motility/invasion by inhibiting EGFRvIII/AKT, EGFRvIII/JAK2/STAT3, and focal adhesion kinase (FAK) signaling pathways respectively. Interestingly, afatinib specifically inhibited EGFRvIII-cMET crosstalk in CSCs, resulting in decreased expression of Nanog and Oct3/4, and in combination with TMZ significantly decreased their self-renewal property in vitro. More interestingly, afatinib and TMZ combination significantly decreased the xenograft growth and progression compared to single drug alone.

**Conclusion:**

Our study demonstrated significant inhibition of GBM tumorigenicity, CSC maintenance in vitro*,* and delayed tumor growth and progression in vivo by combination of afatinib and TMZ. Our results warrant evaluation of this drug combination in EGFR and EGFRvIII amplified GBM patients.

**Electronic supplementary material:**

The online version of this article (10.1186/s13046-019-1264-2) contains supplementary material, which is available to authorized users.

## Background

Glioblastoma (GBM) accounts for 45.6% of malignant brain tumors and is universally fatal [1]. Despite the multimodality treatment options available to newly diagnosed GBM patients including surgical resection, radiotherapy and temozolomide (TMZ)-based concomitant and adjuvant chemotherapy [2], tumor recurrence is inevitable and results in poor median progression-free survival (PFS) (6.9 months) [3]. Scant progress has been made in the last decade to improve survival [4, 5].

Several histologic and cancer genome sequencing studies have revealed deregulation of EGFR and its downstream signaling pathways in GBM [6–8]. Specifically, 30–60% of primary GBM patients carry EGFR amplification [9, 10], and ~ 50–60% of GBM tumors with EGFR amplification also carry constitutively active EGFR variant III (EGFRvIII) [11–13]. In addition to EGFR, mutations in ERBB2/HER2 are also reported in 7–15% of GBM patients [14, 15]. EGFR family members control cell differentiation, proliferation, survival, and migration, while aberrant activation of these receptors results in persistent activation of the PI3K/AKT/mTOR and Ras/Raf/ERK signaling pathways implicated in the development and progression of several tumors including GBM [16]. In addition, EGFR/EGFRvIII signaling maintains GBM cancer stem cells (CSCs) also called side population (SP) [17–19] and control tumor progression, recurrence, and resistance to chemoradiation therapy (CRT) [20, 21]. Though CSCs from many tumors overexpress EGFR [22, 23], GBM CSCs show resistance to anti-EGFR therapies by compensatory upregulation of HER2 and HER3 [24]. In addition, co-activation of other receptor tyrosine kinases (RTKs) has also been implicated in limiting the efficacy of anti-EGFR therapies [25]. Recently, EGFRvIII was reported to cross-activate cMET RTK signaling [25–27], and result in increased growth and enrichment of GBM CSCs [28, 29]. Immunohistochemical analysis also showed increased co-expression of stem cell markers and cMET in GBM patient specimens [28], and patient-derived neurospheres [29]. Furthermore, cMET co-precipitates with EGFR in GBM patient biopsies and mouse xenografts [30]. Interestingly, the addition of either cMET or PDGFRα inhibitor along with erlotinib (a first generation EGFR inhibitor) significantly suppressed GBM cell growth compared to erlotinib alone. These findings highlight the need to develop therapies targeting both EGFR family members and co-activators in GBM cells for effective growth inhibition and prevention of recurrent tumor development. [25].

Afatinib is an FDA-approved, irreversible inhibitor [31] that blocks activation of EGFR, HER2, HER4, and EGFRvIII by irreversibly binding to their ATP binding site [32, 33]. Recent studies have shown a significant increase in the PFS of afatinib-treated non-small-cell lung cancer (NSCLC) patients with EGFR mutation compared to patients treated with pemetrexed plus cisplatin or gemcitabine plus cisplatin [34, 35]. Interestingly, afatinib also significantly improved the PFS of EGFR mutant NSCLC patients with brain metastases [36]. In addition, afatinib significantly increased overall survival (OS) and PFS in lung squamous cell carcinoma patients compared to erlotinib [37]. Though afatinib is shown to cross the blood-brain barrier (BBB) [38], a recent study showed no improvement in non-selected recurrent GBM patients [39]. Intriguingly, afatinib significantly increased OS (six fold) of a patient with recurrent GBM overexpressing EGFR and EGFRvIII [40].

TMZ is a DNA alkylating agent and the standard chemotherapeutic drug for GBM. TMZ in combination with radiation therapy (RT) and adjuvant significantly increased the OS (14.6 months vs 12.1 months) of GBM patients compared to RT alone [3]. Though previous study demonstrated no pharmacokinetic alteration of TMZ upon co-administration with afatinib [39], however, therapeutic efficacy and the molecular mechanism(s) of this combination is still unknown.

We analyzed the efficacy of afatinib and TMZ combination in EGFRvIII-amplified GBM using in vitro and in vivo models. Our study revealed that the combination of afatinib and TMZ synergistically decreased cell proliferation, clonogenicity, invasion, and motility of U87EGFRvIII and U251EGFRvIII cells in vitro and significantly inhibited the growth of U87EGFRvIII orthotopic xenografts in vivo. Our mechanistic studies revealed that afatinib reduces CSCs and tumor growth by inhibiting EGFRvIII-mediated cMET and JAK2/STAT3 pathway activation, enhancing TMZ-induced cytotoxicity.

## Materials and methods

Temozolomide (TMZ) and afatinib were obtained from Sigma-Aldrich (T2577; St. Louis, MO) and Selleck chemicals (S1011; Houston, TX), respectively. The 24 well plate cell culture inserts (#3422) and BioCoat™ Matrigel® invasion chambers (#354480, Corning Incorporated, USA) were used to analyze migration and invasion respectively. All antibodies used in this study are summarized in Additional file 4: Table S1.

### Cell culture

Human GBM cell lines U87MG and U87 cells transfected with either EGFR WT, EGFRvIII or EGFRvIII DK (dead kinase) were a generous gift from Dr. Webster K. Cavenee (University of California San Diego, CA, USA), and U251 cells transfected with EGFR WT or EGFRvIII under the control of tetracycline (Tet)-inducible promoter were gifted by Dr. Amyn A. Habib (University of Texas Southwestern Medical Center, Dallas, TX, USA). All cell lines were cultured as described earlier [41]. Cell line authentication was done by PCR-based STR analysis at the University of Arizona genetics core, Tucson, AZ.

### MTT assay

The cytotoxicity of TMZ- and afatinib-treated GBM cells was measured by MTT assay as described earlier [42] Briefly, U87MG (3 × 10^3^/well) and U87EGFRvIII (2 × 10^3^/well) cells were seeded in a 96-well plate overnight and incubated with drugs or vehicle [(0.02% dimethyl sulfoxide (DMSO)] for 24–72 h at 37 °C. MTT (5 mg/ml) was added and cells were incubated for 4 h at 37 °C. Formazan crystals were dissolved in DMSO, plates were read at 570 nm using a Spectra MAX 190 plate reader (Molecular Devices, LA, USA), and viable cells were calculated [42].

### Combination index (CI)

The combination effects or index (CI) of TMZ and afatinib was calculated using CompuSyn software as described earlier [43]. A CI < 1 means synergistic effect, whereas CI equal to 1 or > 1 indicate additive and antagonist effects, respectively.

### Clonogenic survival and soft agar assay

Both the colony formation and soft agar assays were done as described earlier [42]. Briefly, 2000 cells/well were seeded in a six-well plate with complete media. After overnight incubation, cells were treated with either 0.02% of DMSO, 1 μM of afatinib, 25 μM of TMZ or combination for 48 h. After washing with PBS, cells were allowed to grow in 2% media for 12 days, and colonies were fixed in methanol and stained with crystal violet solution. Colonies were dissolved in 10% acetic acid [44] and absorbance was measured at 570 nm using a Spectra MAX 190 plate reader.

For soft agar assay, plates were coated with 0.5% agarose, and 5000 cells containing DMEM with 20% FBS and 0.25% agarose were seeded on the top of base agar. The next day, cells were treated with vehicle, 1 μM of afatinib, 25 μM of TMZ or combination for 30 days with fresh media change every third day. Colonies were stained with crystal violet solution (0.1% crystal violet in 20% methanol) and counted [42]. Colony sizes of ≥70 μm was considered as big colonies [45].

### Cell cycle analysis and SA-β-gal staining

The effect of afatinib and TMZ on the cell cycle was analyzed by flow cytometry as described earlier [46]. Quantitative in situ senescence-associated β-galactosidase (SA-β-gal) staining was done as described earlier [47]. Images were taken using light microscopy, and multiple representative areas (*n* = 7) were randomly selected for the quantification of SA-β-gal positive cells.

### Migration and invasion assay

The transwell migration and invasion assays were done as described earlier [42]. Briefly, after treating U87MG and U87EGFRvIII cells with drugs for 48 h, 2.5 × 10^4^ and 5.0 × 10^4^ cells each were re-suspended in 100 μl serum-free DMEM medium with TMZ (25 μM), afatinib (1 μM), or combination and applied on the upper chamber of non-coated and Matrigel-coated transwell chambers for motility and invasion, respectively. The bottom chamber was filled with 600 μl DMEM containing 10% FBS. After 16 h of incubation at 37 °C in a CO_2_ incubator, non-migrated cells in the upper chamber were removed using a cotton swab and migrated/invaded cells were stained with Diff-Quick® cell stain kit (Dade-Behring Inc., Newark, DE, USA) and counted using Image J software.

### Side population assay

Flow cytometry was used to analyze the SP/CSCs as described previously [48]. Briefly, 1 × 10^6^ cells/ml of 10% FBS containing DMEM were incubated with 5 μg/mL Hoechst 33342 (AnaSpec Inc., Fremont, CA, USA) for 60 min at 37 °C. CSCs were sorted by FACS analysis using LSR II Green (BD Biosciences). Verapamil (50 μM/ml), an ABC transporters inhibitor, was used as a control to identify CSCs.

### Neurosphere assay

Cells were cultured in DMEM-F12 (Invitrogen-Life Technologies), together with basic fibroblast growth factor (bFGF) (10 ng/mL), epidermal growth factor (EGF) (20 ng/mL), leukemia inhibitory factor (LIF) (10 ng/mL) and 10% knockout serum (all from Sigma). Two-hundred μL of the medium containing 2000 cells/well were plated in 96-well low attachment culture plates. Those spheres with a diameter of ≥100 μm within each well were counted after 10 days of culture. Images were captured using a Carl Zeiss microscope.

### Intracranial injection and bioluminescence imaging

All animal experiments were carried out according to protocols approved by the Institutional Animal Care and Use Committee (IACUC). Intracranial injection into four to six-week-old athymic nude mice was done as described earlier with slight modifications [49]. Briefly, mice were anesthetized by intraperitoneal (i.p.) injection of ketamine and xylazine and immobilized on a stereotactic frame (Stoelting Co, IL, USA). A Hamilton syringe with a 26-gauge needle was inserted at 1-mm dorsal and 2-mm lateral to the bregma to a depth of 3.5-mm and then pulled back 0.5-mm to allow space for tumor cells. Following this, U87EGFRvIII luciferase-transfected cells (2 × 10^4^ in 2 μl of PBS) were injected at an injection speed of 0.5 μl/min. After 5 days, luciferase substrate was injected (i.p. 100 μl of 5 mM CycLuc1) and tumor growth was measured using bioluminescence imaging after 10 min in an IVIS spectrum, Caliper life sciences (PerkinElmer, MA, USA). Tumor volume (photon flux) of the mice was measured using Living Image® software, PerkinElmer, MA, USA. After 5 days of tumor implantation, based on tumor volume, animals were randomized into four groups and treated with vehicle, TMZ (25 mg/kg BW), afatinib (10 mg/kg BW) or combination for 5 days a week by oral gavage for 30 days (treatment start date was considered as day 1). Vehicle-treated and afatinib-treated animals were sacrificed when they were very weak. TMZ and combination group animals were euthanized after 30 days.

### Statistical analysis

Each experiment was repeated at least three times and data were expressed as mean values ± SD. The student t-test and ANOVA were used to determine significant differences between the groups with *p*-values <0.05 considered statistically significant.

## Results

### Afatinib and TMZ combination differentially inhibit the proliferation and clonogenic survival of EGFR and EGFRvIII expressing GBM cells

**Fig. 1:**
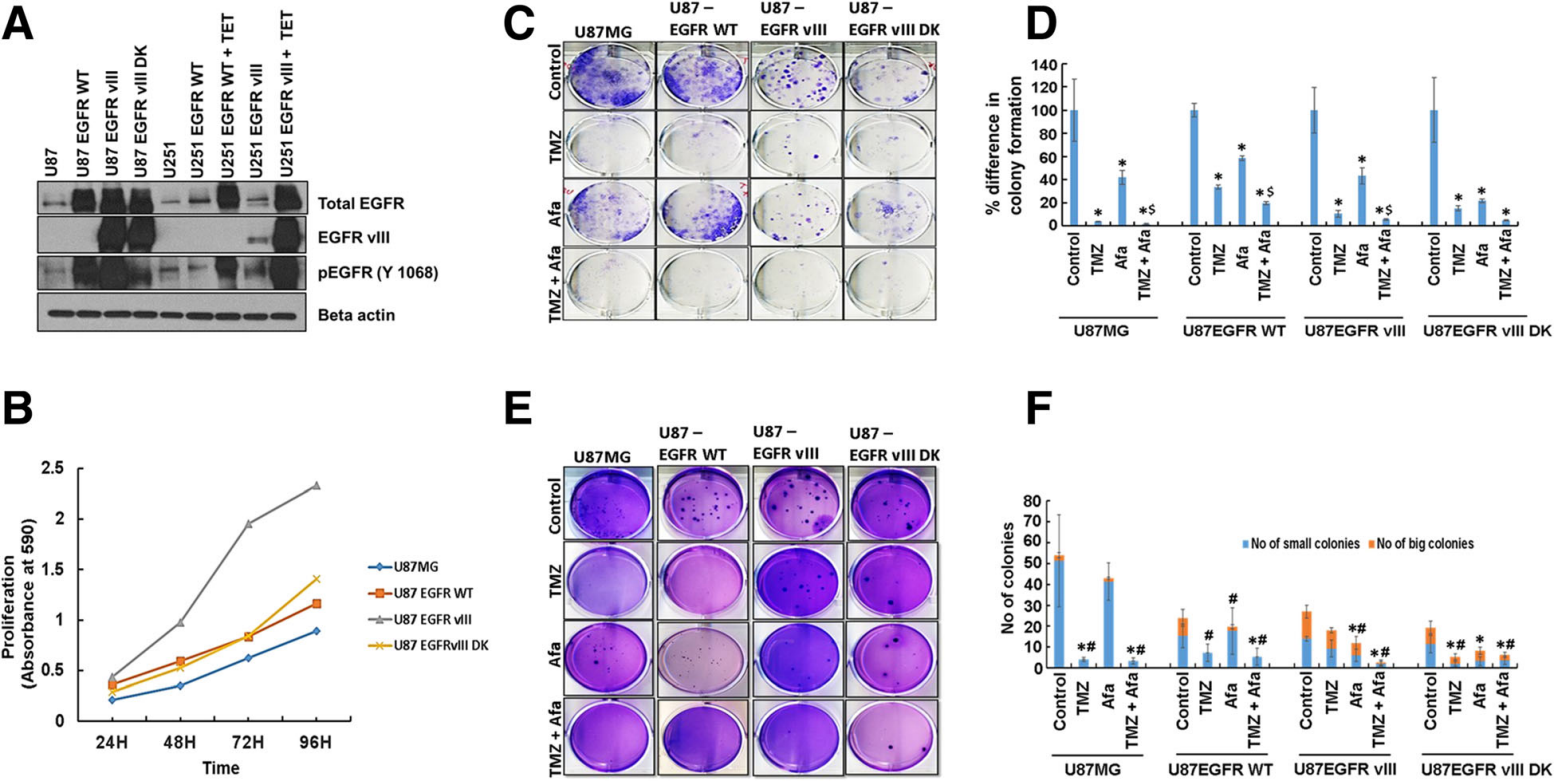
Afatinib and TMZ combination differentially inhibit proliferation and clonogenic survival of EGFR and EGFRvIII expressing GBM cells. **a, b** EGFR and EGFRvIII expression differentially effect cell proliferation. **a** U87MG, U87EGFR WT, U87EGFRvIII, U87EGFRvIII DK, U251, U251EGFR WT (Tet-inducible system) and U251EGFRvIII (Tet-inducible system) cell lysates were analyzed for EGFR (full length), EGFRvIII and pEGFR (Tyr-1068). **b** The graph shows increased proliferation rate of U87EGFRvIII cells compared to U87MG, U87EGFR WT, and U87EGFRvIII DK cells. Tet - tetracycline. **c**, **d** U87MG, U87EGFR WT, U87EGFRvIII and U87EGFRvIII DK cells were seeded and treated as specified for 48 h. After washing with PBS, cells were allowed to grow for 12 days in 2% media. Colonies formed were fixed with methanol, stained with crystal violet solution, dissolved in 10% acetic acid and absorbance was measured at 570 nm. The graph shows the mean (± SD) percentage of colony formation. The experiment was repeated three times (*^$^ *P* ≤ 0.05); * significant compared to control; $ significant compared to TMZ. **e**, **f** Afatinib and TMZ combination decreases the anchorage-independent growth of TMZ-resistant EGFRvIII GBM cells. U87MG, U87 EGFR WT, U87EGFRvIII and U87EGFRvIII DK cells (5 × 10^3^) were seeded with 0.25% agarose on the top of the 0.5% base agar. After overnight incubation, cells were incubated with TMZ (25 μM) or afatinib (1 μM) or combination of both for a month. The 0.1% crystal violet stained colonies were counted (*n* = 3). The graph shows mean (± SD) number of small (blue) and big colonies (orange). (* P ≤ 0.05); * number of small colonies, significant compared to control; ^**#**^ number of big colonies, significant compared to control

EGFRvIII is known to increase the proliferation, survival and modulate therapeutic response of cancer cells [50]. We validated the expression of EGFR, EGFRvIII and EGFRvIII DK in U87MG and U251 cells by western blot analysis (Fig. 1a), and analyzed the proliferation rates by MTT assay. In concordance with the previous report [51], we observed significantly increased (p < 0.0001) proliferation of U87EGFRvIII cells compared to parental U87MG, EGFR and EGFRvIII DK over expressed cells (Fig. 1b). To analyze the cytotoxic effects of TMZ and afatinib on GBM, U87MG and U87EGFRvIII cells were treated with varying concentrations of TMZ (10–500 μM) or afatinib (0.25–5.0 μM) for 48–72 h and analyzed by MTT assay. A dose-dependent decrease in the viability of GBM cells was observed following TMZ and afatinib treatment. The inhibitory concentration of 25% (IC_25_) values was approximately 25 μM and 300 μM for TMZ, and 2 μM and 1 μM of afatinib for U87MG and U87EGFRvIII cells, respectively. Recently, a pharmacokinetic analysis on 35 GBM patients revealed that approximately 10–25 μM concentrations of TMZ reaches Cerebrospinal Fluid (CSF) [52, 53] and therefore to mimic the in vivo conditions, we used 25 μM of TMZ and 1 μM afatinib for all our in vitro experiments. Accordingly, our CI plot revealed an additional and near synergistic decrease in cell viability upon combining afatinib with TMZ in U87EGFRvIII cells (Additional file 1: Figure S1 A-F).

Colony formation assay revealed that both afatinib and TMZ significantly decreased the clonogenicity of GBM cells U87MG, U87EGFR WT, U87EGFRvIII, and U87EGFRvIII DK compared to vehicle-treated control cells (Fig. 1c, d). TMZ inhibited colony formation more than afatinib in U87MG, U87EGFR WT, and U87EGFRvIII DK cells, while U87EGFRvIII cells were relatively resistant (Fig. 1c, d). However, combining afatinib with TMZ abolished the colony forming ability of U87EGFRvIII cells (Fig. 1c, d). The percentage decreases in colony formation of U87MG cells were 96.3 ± 0.3, 58 ± 6.2, 98.4 ± 0.7 for TMZ (*p* = 0.01), afatinib (*p* = 0.03) and TMZ plus afatinib (*p* = 0.01) compared to vehicle-treated control, respectively. The percentage decreases in colony formation of U87 EGFR WT were 66.5 ± 1.8, 41.4 ± 1.9 and 80.5 ± 1.2 for TMZ, afatinib and combination group compared to control (*p* = 0.001), respectively. U87EGFRvIII formed bigger colonies than U87MG, U87EGFR WT, and U87EGFRvIII DK cells. Similarly, the mean percentage decreases in colonies formed by U87EGFRvIII were 89.6 ± 3.0, 56.6 ± 7.0 and 94.5 ± 0.6 for TMZ-, afatinib- and combination-treated groups compared to control, respectively. In addition, afatinib plus TMZ significantly decreased EGFRvIII colonies when compared to either TMZ (*p* = 0.048) or afatinib (*p* = 0.005) alone. The percentage decreases in colony formation of U87EGFRvIII DK were 85.0 ± 2.3, 78.3 ± 1.5 and 95.3 ± 0.3 for TMZ (*p* = 0.02), afatinib (p = 0.02) and combination group (p = 0.01) when compared to control, respectively (Fig. 1d)**.** Overall, combination treatment significantly decreased U87MG, U87EGFR WT, and U87EGFRvIII colony growth when compared to TMZ alone (*p* = 0.01). Similar results were observed for U251 and U251EGFRvIII GBM cells (data not shown).

We analyzed whether afatinib could block the anchorage-independent growth of U87EGFRvIII cells, since EGFRvIII plays an important role in cancer cell proliferation [51], and anchorage-independent growth predicts in vivo tumorigenicity [54]. TMZ significantly decreased the colony size, as well as the number of colonies in U87MG, U87EGFR WT, and U87EGFRvIII DK, while TMZ had less effect on EGFRvIII cells. (Fig. 1e). TMZ alone or in combination with afatinib significantly decreased the number of U87MG small colonies (*p* = 0.03) and big colonies (*p* = 0.02) as well. TMZ and afatinib individually significantly decreased the number of U87EGFR WT big colonies (*p* = 0.05) while TMZ and afatinib combined significantly decreased both the number of small colonies (p = 0.03) and big colonies (p = 0.03). Afatinib or combination treatment significantly decreased the number of U87EGFRvIII small [(afatinib (p = 0.02); combination (p = 0.01)] and big colonies (afatinib (p = 0.02); combination (p = 0.01)). TMZ or afatinib or combination treatment significantly decreased the number of U87EGFRvIII DK number of small colonies (p = 0.03), while TMZ and combination treatment significantly decreased the number of big colonies [TMZ (*p* = 0.048); combination (*p* = 0.038)]. Overall, combination therapy with afatinib completely abrogated anchorage-independent growth of GBM cells regardless of the activation status of EGFR (Fig. 1f).

### Afatinib inhibits EGFR activation in GBM cells

**Fig. 2:**
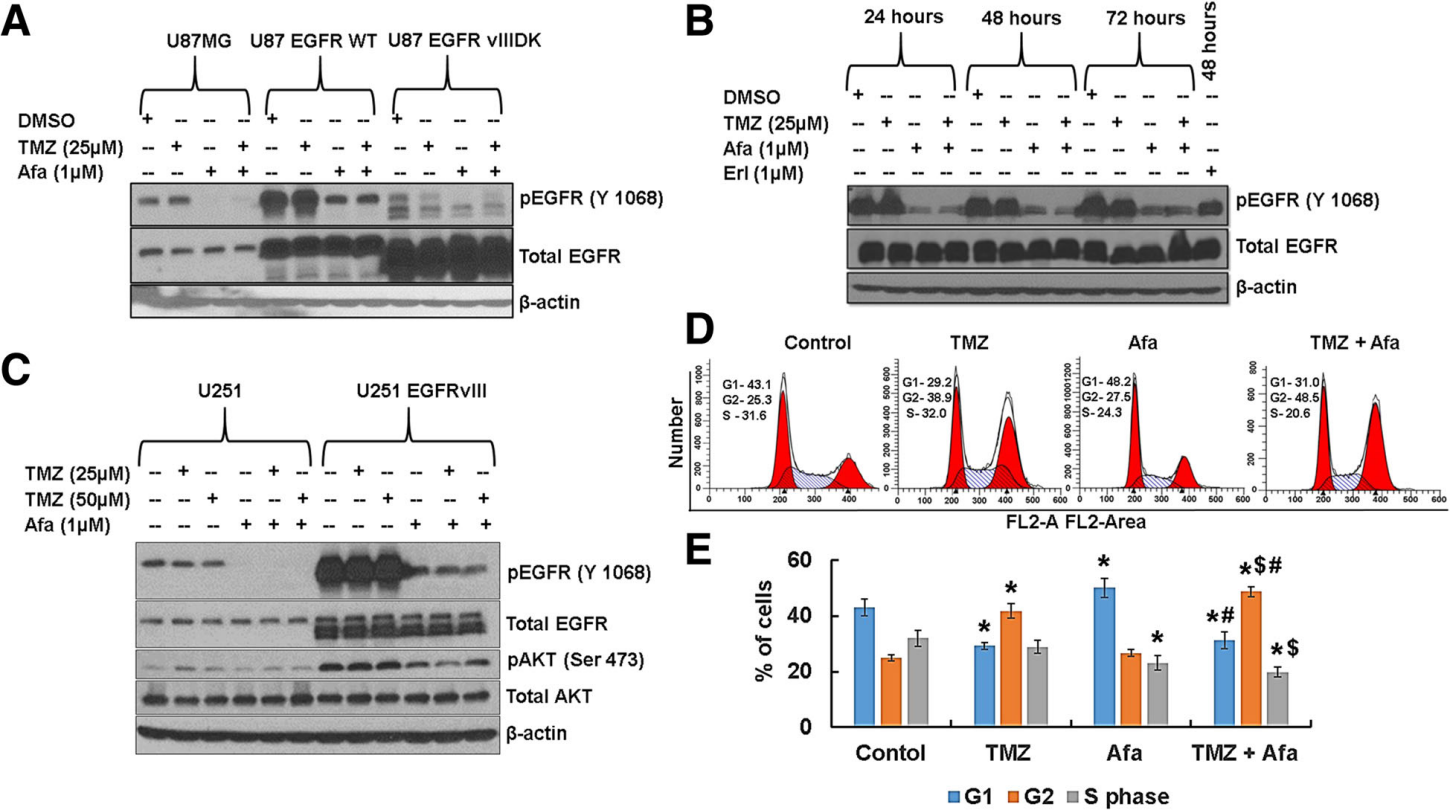
Afatinib inhibits EGFR and EGFRvIII activation and augments G_2_/M arrest with TMZ. **a** U87MG, U87EGFR WT and U87EGFRvIII DK cells were incubated with TMZ (25 μM), afatinib (1 μM) or combination of drugs for 48 h, and cell lysates were analyzed for pEGFR (Tyr-1068) by western blot analysis. β-actin serves as a loading control. **b** U87EGFRvIII cells were treated with TMZ, afatinib, erlotinib or combination of TMZ and afatinib and analyzed for pEGFR (Tyr-1068). **c** U251 and U251EGFRvIII cells were treated with afatinib, TMZ or combination for 48 h. Cell lysates were analyzed for pEGFR (Tyr-1068) and pAKT (Ser-473) by western blot analysis. **d** U87EGFRvIII cells were synchronized with double thymidine block and treated with TMZ (25 μM), afatinib (1 μM) or combination of drugs for 48 h. Cells were trypsinized, fixed with 70% ethanol, stained with Telford reagent and analyzed by flow cytometry. **e** The bar diagram shows the mean (± SD) percentage of distribution of the cells (*^$#^ P ≤ 0.05); * significant compared to control; $ significant compared to TMZ; # significant compared to afatinib (n = 3)

We examined the effect of afatinib alone or in combination with TMZ on EGFR activation by immunoblotting. Afatinib treatment significantly inhibited EGFR activation (phosphorylated EGFR) in U87MG, U87EGFR WT, U87EGFRvIII, and U87EGFRvIII DK, whereas TMZ had no effect (Fig. 2a, b). Interestingly, we observed that afatinib inhibited pEGFR longer and more potently than erlotinib (first-generation EGFR inhibitor) in EGFRvIII cells (Fig. 2b). Afatinib and erlotinib had no effect on total EGFR (Fig. 2b). Afatinib had similar inhibitory effects on EGFR activation in GBM cell lines U251 and U251EGFRvIII (Fig. 2c).

### Afatinib and TMZ combination induces cell cycle arrest in EGFRvIII GBM cells

TMZ is known to induce G_2_/M arrest and inhibit U87MG GBM cell proliferation [55]. U87EGFRvIII cells are more resistant to the cytotoxic drug cisplatin than U87MG cells [56]. We analyzed the effect of afatinib and TMZ on the cell cycle in U87EGFRvIII cells by flow cytometry. Afatinib and TMZ significantly induced G1 (*p* = 0.001) and G_2_/M arrest (p = 0.001), respectively, while decreasing the proportion of EGFRvIII cells in S-phase compared to vehicle-treated control cells (Fig. 2d, e). We further observed that the combination of afatinib and TMZ significantly reduced the percentage of S-phase cells (*p* = 0.001), but increased the proportion of G_2_/M-arrested (p = 0.001) U87EGFRvIII cells (Fig. 2d, e).

The G_2_/M phase arrest may either allow cells to repair damaged DNA and proliferate or induce cell death by apoptosis, senescence, or mitotic catastrophe [57]. As we did not observe EGFRvIII cells in sub-G_0_ (apoptosis) after drug treatment, we analyzed senescence-associated β-galactosidase (SA-β-gal) activity, as a marker for cellular senescence. TMZ and afatinib combination treatment led to significantly increased SA-β-gal-stained EGFRvIII cells (*p* = 0.0001) (24 ± 4.5%) as compared with TMZ, afatinib or treatment control cells (6.6 ± 1.6%, 10 ± 2%, and 2 ± 1%, respectively) (Additional file 2: Figure S2 A-B).

### Afatinib and TMZ combination decreases GBM cell migration and invasion

**Fig. 3:**
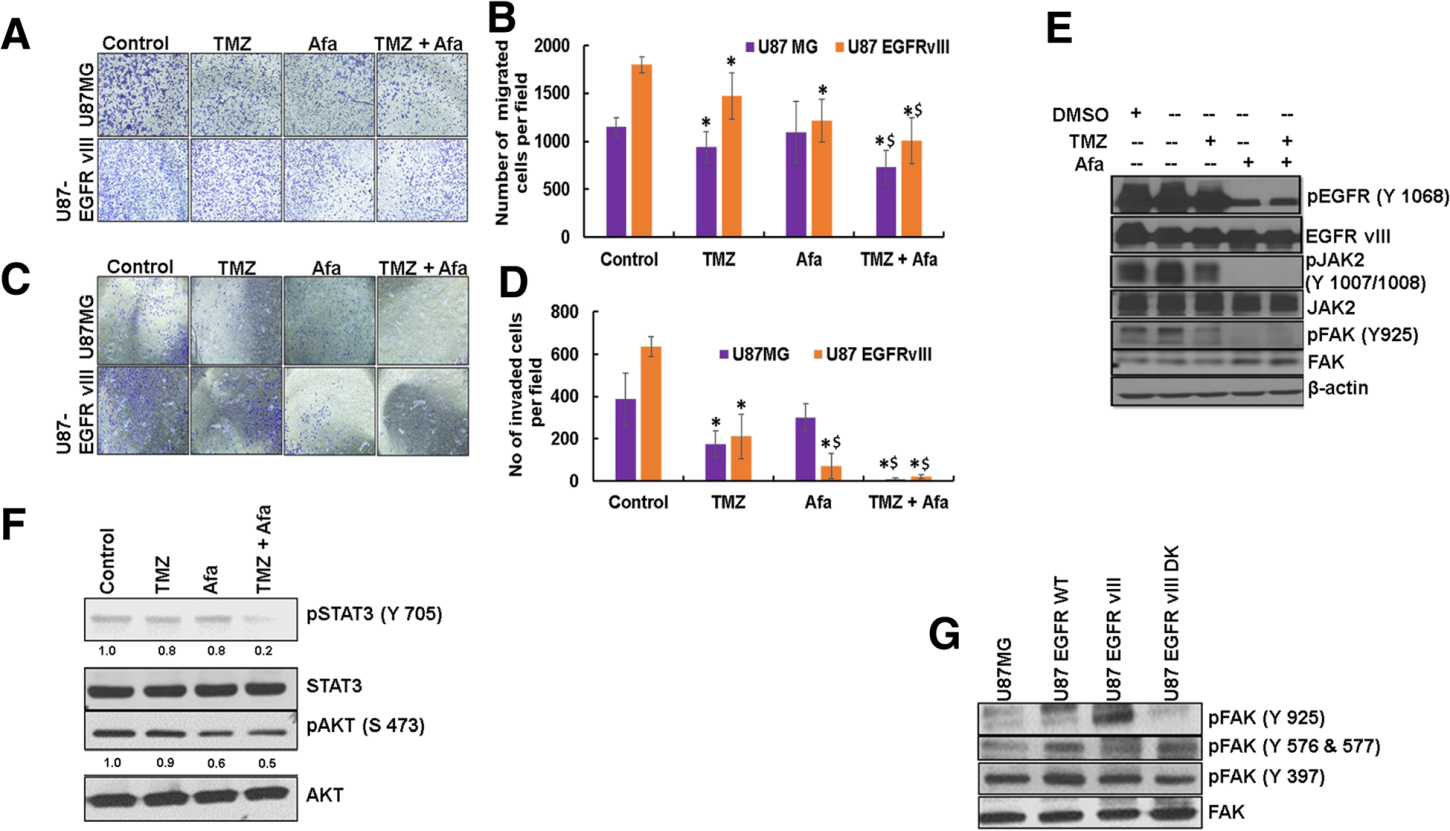
Afatinib and TMZ combination decreases migration and invasion of U87MG and U87 EGFRvIII cells. **a**, **b** U87MG and U87EGFRvIII cells (2.5 × 10^4^) were incubated with TMZ (25 μM), afatinib (1 μM) or combination of drugs for 48 h. Non-migrated cells in the upper chamber were removed with a cotton swab, and the migrated cells were stained and counted. Representative images are shown (10X magnification). The bar graph shows the mean (± SD) percentage of migrated cells. Experiments were repeated three times and 5 random fields were chosen for quantification (*^$^ P ≤ 0.05); * significant compared to control; $ significant compared to TMZ. **c**, **d** Afatinib alone or in combination with TMZ decreases the invasion of U87EGFRvIII cells. U87MG and U87 EGFRvIII cells (5.0 × 10^4^) were incubated with TMZ (25 μM), afatinib (1 μM) or combination of drugs for 48 h. Non-invaded cells in the upper chamber was removed with a cotton swab, and the invaded cells were stained and counted. The bar graph shows the mean (± SD) percentage of invaded U87MG and U87EGFRvIII cells. Experiments were repeated three times (*^$^ P ≤ 0.05); *significant compared to control; $ significant compared to TMZ. **e**, **f** Afatinib inhibits EGFRvIII-mediated JAK2/STAT3 and FAK signaling. U87EGFRvIII cells were treated with TMZ (25 μM), afatinib (1 μM) or combination for 48 h, and lysates were analyzed for pEGFR (Tyr-1068), pJAK2 (Tyr-1007/1008), pSTAT3 (Tyr-705), pFAK (Tyr-925) and pAKT (Ser-473) by western blot analysis. **g** EGFRvIII kinase domain mediates FAK (Tyr-925) activation. U87, U87 EGFR WT, U87EGFRvIII and U87EGFRvIII DK cell lysates were analyzed for pFAK (Tyr-925), pFAK (Tyr-576/577) and pFAK (Tyr-397)

GBM is locally aggressive and invades the perivascular regions [58]. We analyzed the effect of afatinib and TMZ on U87MG and U87EGFRvIII GBM cell migration and invasion. Afatinib alone or in combination with TMZ significantly decreased the migration of U87EGFRvIII cells compared to U87 cells (Fig. 3a, b). Afatinib alone had no significant effect on U87MG cells (Fig. 3 a, b). The number of U87MG-migrated cells decreased significantly in the TMZ (*p* = 0.02) and combination (*p* = 0.001) groups; TMZ vs TMZ plus afatinib (*p* = 0.04)). Similarly, TMZ and combination treatment significantly decreased the migration of U87EGFRvIII (control vs any treatment (p = 0.02); TMZ vs TMZ plus afatinib (p = 0.02)). TMZ (*p* = 0.03) and combination (p = 0.02) treatments significantly decreased the number of invasive U87MG cells when compared to control (Fig. 3c, d)**.** The number of invasive U87EGFRvIII cells decreased significantly in treated cells when compared to control [TMZ (*p* = 0.005); afatinib (*p* = 0.01)]; combination (*p* = 0.0002)). Further, both in U87MG and U87EGFRvIII, combination treatment significantly decreased the number of invasive cells when compared to TMZ alone [U87MG (p = 0.001); U87EGFRvIII (p = 0.03)].

EGFRvIII is known to activate JAK2/STAT3 signaling, and its inhibition has been shown to decrease invasion both in vitro and in vivo [59]. STAT3 activation is found to be higher in GBM than in low-grade astrocytoma, and it is co-expressed with EGFR in GBM [60]. Inhibition of JAK2/STAT3 signaling sensitized U87EGFR WT and U87EGFRvIII cells to the anti-EGFR drug gefitinib [60]. Interestingly, our results showed that TMZ and afatinib together significantly decreased STAT3 signaling as well as cell survival AKT signaling (Fig. 3f). In addition, EGFR also mediates FAK phosphorylation (Y925) through Src, resulting in cytoskeletal reorganization and increased cell motility/invasion [59, 61–63]. To investigate whether the kinase activity of EGFRvIII mediates FAK Y925 phosphorylation, we analyzed FAK activation (pFAK-Y925) in U87MG, U87EGFR WT, U87EGFRvIII, U87EGFRvIII DK and U251EGFRvIII cells. As shown in Fig. 3g and (Additional file 3: Figure S3), pFAK (Y925) signaling was specific to EGFRvIII, as no activation was observed in U87MG and U87EGFRvIII DK cells. Importantly, afatinib treatment resulted in complete downregulation of both pJAK2 and pFAK in U87EGFRvIII cells (Fig. 3e), suggesting their involvement in EGFRvIII-mediated GBM cell invasion. No changes in other FAK phosphorylation sites (Y397 and Y576/577) were observed in EGFRvIII cells (Fig. 3g).

### Afatinib reduces SP/CSCs by inhibiting EGFRvIII-cMET cross-activation

**Fig. 4:**
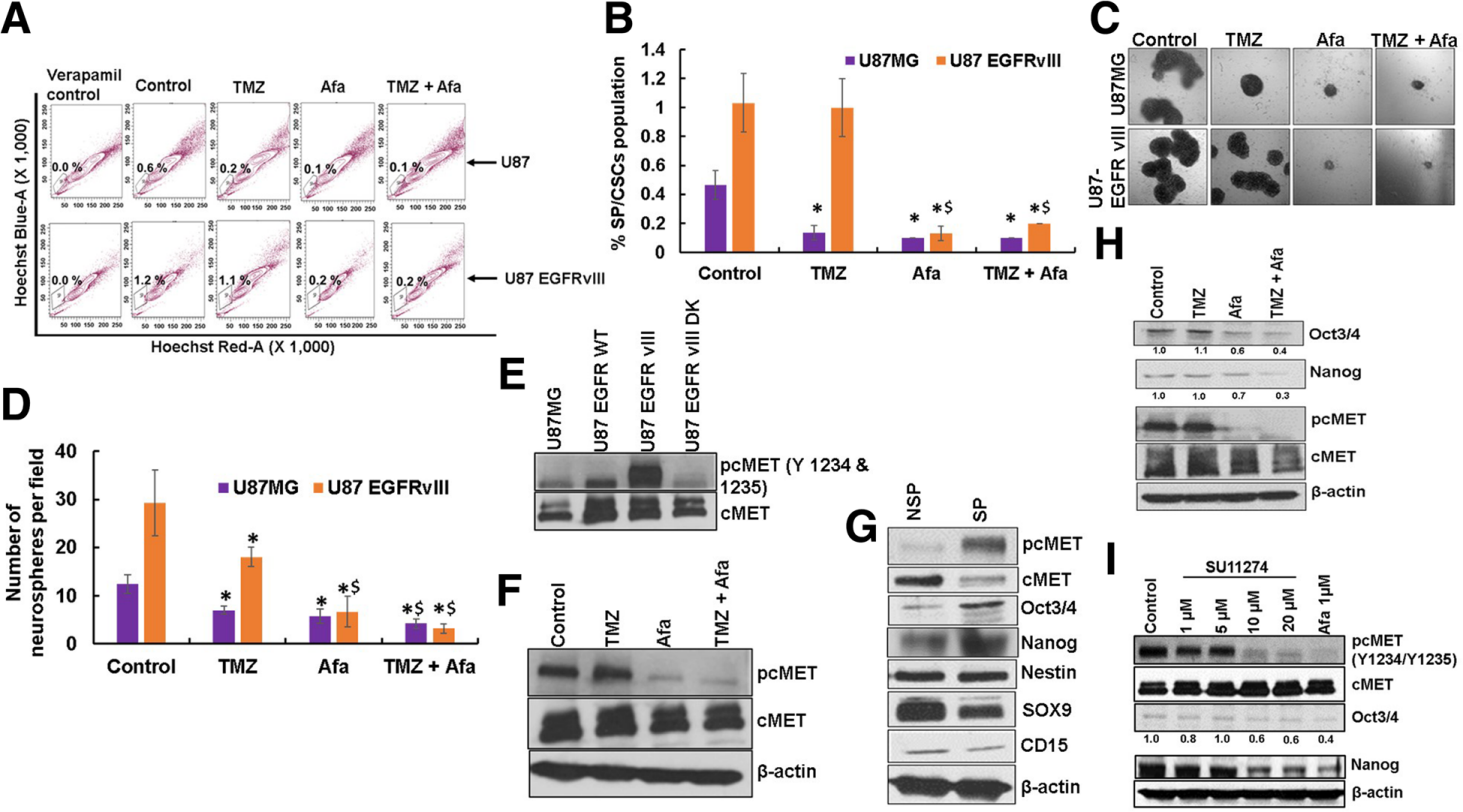
Afatinib inhibits EGFRvIII-cMET signaling crosstalk in SP/CSCs cells. **a**, **b** Afatinib reduces TMZ-resistant U87EGFRvIII SP/CSCs. U87MG and U87EGFRvIII cells treated with either TMZ (25 μM), afatinib (1 μM) alone or combination for 48 h were trypsinized, stained with Hoechst 33342 (5 μg/ml) and analyzed for CSCs and NSP cells using flow cytometry. The bar diagram shows the mean (± SD) percentage of SP/CSCs from U87MG and U87EGFRvIII (n = 3). **c**, **d** Afatinib decreases U87EGFRvIII-mediated self-renewal properties of CSCs. U87MG and U87EGFRvIII cells treated with TMZ (25 μM), afatinib (1 μM), or combination for 48 h were plated (2 × 10^3^ cells/well in 100 μl of stem cell medium) on a 96-well ultra-low attachment plate. Neurosphere/tumor spheres formed after 10 days were quantified and photographed (X20 magnification). The graph shows the mean (± SD) number of tumor spheres formed by U87MG and U87EGFRvIII cells (*n* = 4). (*^$^ P ≤ 0.05); * significant compared to control; $ significant compared to TMZ. **e** U87, U87EGFR WT, U87EGFRvIII and U87EGFRvIII DK cell lysates were analyzed for pcMET (Tyr-1234/1235) by western blot analysis. **f** U87EGFRvIII cells were treated with either TMZ (25 μM), afatinib (1 μM), or combination for 48 h and analyzed for pcMET (Tyr-1234/1235). **g** Expression of Nanog, Oct3/4 (self-renewal marker) and pcMET (Tyr-1234/1235) were analyzed in SP/CSCs and NSP cells by western blot analysis. **h** U87EGFRvIII SP cells were treated with TMZ (25 μM), afatinib (1 μM), or combination for 48 h and analyzed for pcMET (Tyr-1234/1235) and stemness markers Nanog and Oct3/4 by western blot analysis. **i** U87EGFRvIII cells were treated with varying concentrations of the cMET specific inhibitor, SU-11274 (1 – 20 μM) or afatinib (1 μM) for 48 h and lysates were analyzed for pcMET (Tyr-1234/1235), Nanog and Oct3/4

Cancer stem cells are highly resistant to CRT and play a major role in tumor recurrence [20, 21]. We observed a significantly higher proportion of CSCs in U87EGFRvIII cells compared to U87MG cells (*p* = 0.03). While afatinib significantly decreased the percentage of CSCs in both U87MG and U87EGFRvIII cells (*p* = 0.02) (Fig. 4a, b), TMZ decreased CSCs in only U87MG cells, (Fig. 4a, b). The average percentages of CSCs in U87EGFRvIII GBM cells were 1.03 ± 0.2, 1.0 ± 0.2, 0.13 ± 0.05 and 0.2 ± 0 in control, TMZ, afatinib and combination groups, respectively.

We further validated our results using an in vitro clonogenic (neurosphere) assay. We observed a significantly higher (*p* = 0.01) number of neurospheres formed by U87EGFRvIII CSCs cells (29 ± 7) than U87MG CSC cells (13 ± 2) (Fig. 4c, d). TMZ significantly decreased the self-renewal properties of U87MG CSCs (*p* = 0.003). We also observed that U87EGFRvIII CSCs were relatively resistant to TMZ compared to U87MG CSCs; however, combined afatinib and TMZ significantly decreased the number of neurospheres in both cell lines (*p* = 0.001) (Fig. 4c, d). The average number of U87EGFRvIII CSC neurospheres per field was 29 ± 7, 18 ± 2, 7 ± 3 and 3 ± 1 in control, TMZ, afatinib, and combination treatment groups, respectively (Fig. 4d).

cMET signaling promotes and enriches GBM CSCs [28, 29]. cMET activation was seen in stem cells of GBM patient specimens [28] as well as in several GBM patient-derived neurospheres [29]. We observed increased activation of cMET in U87EGFRvIII cells compared to U87MG (Fig. 4e). Furthermore, afatinib significantly diminished cMET activation in U87EGFRvIII cells (Fig. 4f). To determine if EGFRvIII mediates CSC maintenance through cMET activation in GBM, we analyzed the expression of various stemness markers in CSCs and non-side population (NSP) cells isolated from U87EGFRvIII cells. We observed enrichment of stemness markers Nanog and Oct3/4 and increased cMET activation in CSCs compared with NSP cells. Surprisingly, CSCs expressed lower SOX9 and CD15 levels, and no change in expression of nestin was observed (Fig. 4g). Interestingly, afatinib treatment significantly decreased cMET activation as well as stemness of U87EGFRvIII CSCs, while TMZ only showed no effect (Fig. 4h). To further validate that cMET signaling conserves stemness in GBM, we treated U87EGFRvIII cells with cMET inhibitor SU11274. Our results showed a dose dependent decrease in the expression of phosphorylated/active cMET (pcMET-Y1234/1235) and decreased expression of stemness marker Nanog and Oct3/4 (Fig. 4i).

### Afatinib and TMZ combination prevents tumor growth in vivo

**Fig. 5:**
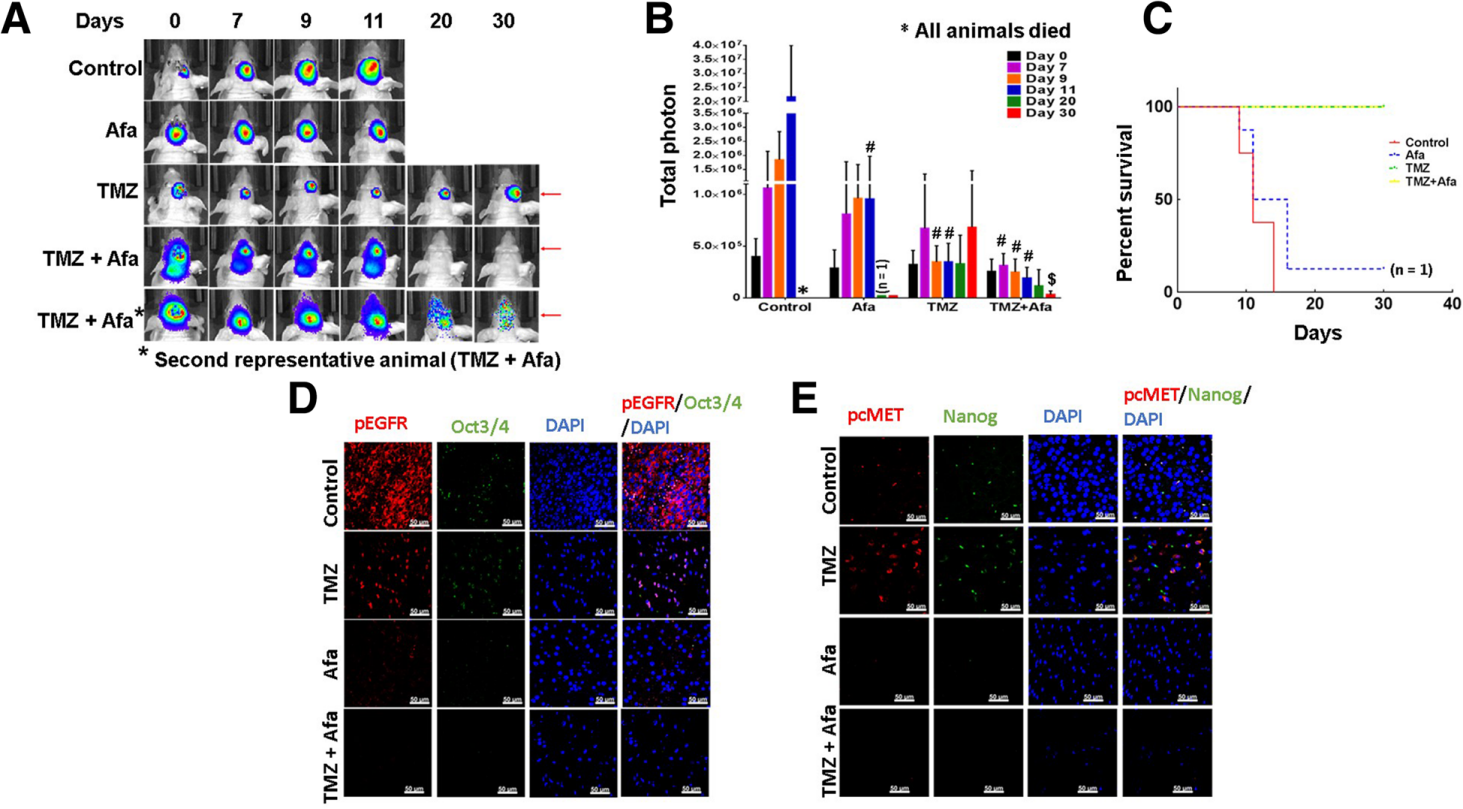
Afatinib and TMZ combination reduces tumor burden in vivo*.* **a**, **b** U87EGFRvIII luciferase transfected cells (2 × 10^4^ in 2 μl of PBS) were intracranially injected into 4–6 week’s old mice using a stereotactic frame. After 5 days, mice were randomized into 4 groups and treated with vehicle (*n* = 8), TMZ (25 mg/kg BW) (*n* = 7), afatinib (10 mg/kg BW) (n = 8) or combination (*n* = 5) for 5 days a week by oral gavage. Animals treated with TMZ alone or in combination with afatinib survived longer and were sacrificed after 30 days. The tumor volume (total photon count) was measured using IVIS imaging on days 0, 7, 9, 11, 20 and 30. (^**$, #**^ *P* ≤ 0.03); $ significant compared to TMZ; # significant compared to vehicle control (**c**) Kaplan Meier survival curve analysis showing effect of control, afatinib, TMZ or combination on OS of EGFRvIII orthograft mice. **d**, **e** Confocal microscopy showing expression of (**d**) pEGFR (Tyr-1068) (red staining), Oct3/4 (green staining) and (**e**) pcMET (Tyr-1234/1235) (red) and Nanog (green) images in U87EGFRvIII tumor xenografts

To analyze the effect of afatinib and TMZ in vivo, U87EGFRvIII luciferase cells were intracranially injected into athymic mice, and treatments were administered once tumors developed (Fig. 5a). We observed that afatinib as a monotherapy neither inhibited tumor growth significantly nor improved the OS of the animal. Only one of eight mice survived until the end of the study (30 days). (Figs. 5C & S 2C). Although TMZ treatment alone initially decreased tumor growth and increased animal survival, tumors progressed in 60% of the mice (Fig. 5b). By contrast, the combination of afatinib and TMZ significantly inhibited tumor growth (*p* = 0.03) after day 30 of treatment compared to TMZ alone. Furthermore, none of the animals exhibited tumor burden in the combination group (Fig. 5a-c), suggesting the superior efficacy of this therapy in GBM in vivo.

**Fig. 6:**
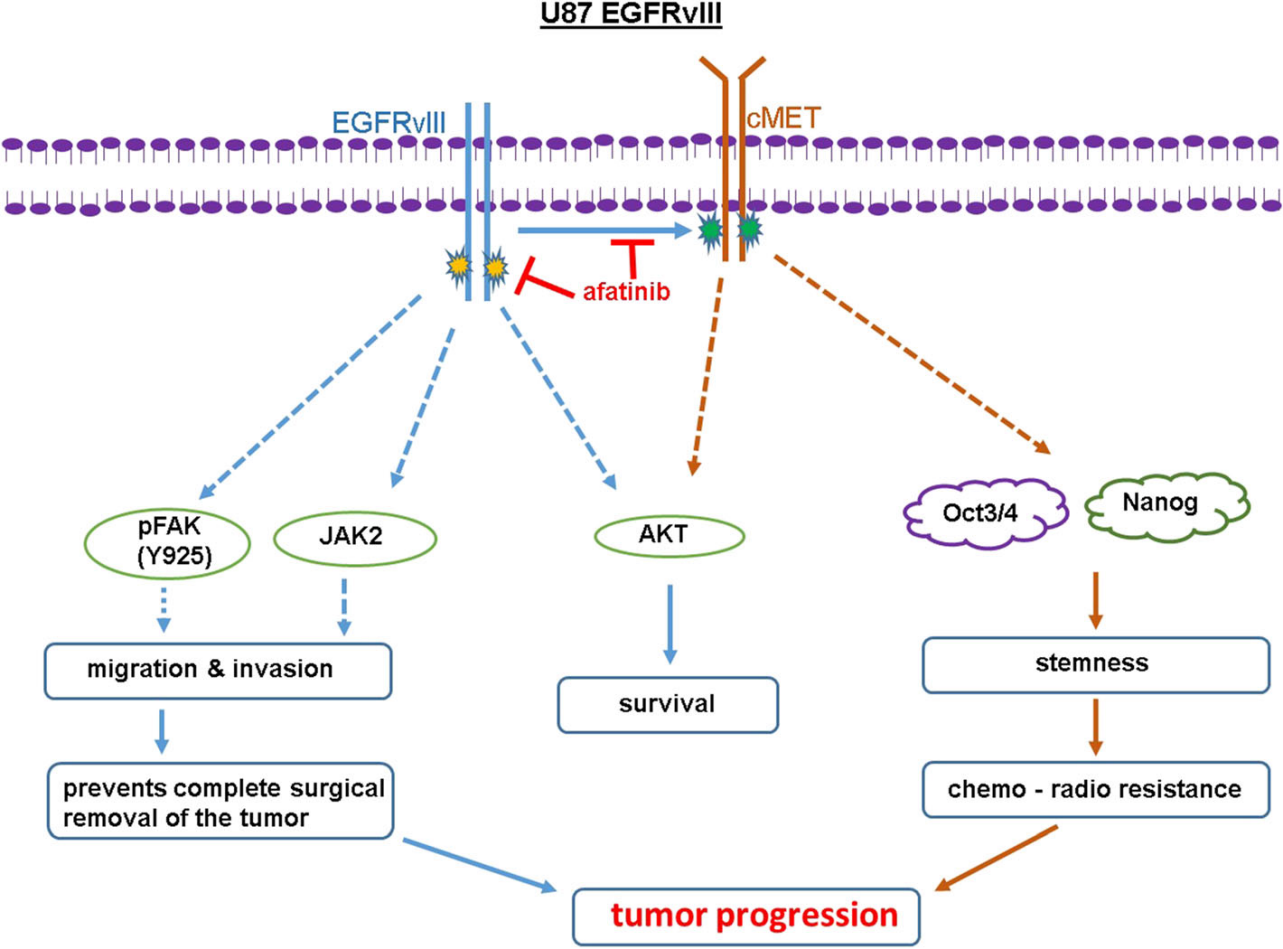
Possible mechanism of afatinib-TMZ combination therapy in GBM. TMZ targets the differentiated proliferating GBM cells but fails to eradicate slow-growing CSCs and result in tumor progression. Afatinib decreases proliferation of U87EGFRvIII cells by inhibiting EGFRvIII/AKT signaling, cell migration and invasion by inhibiting EGFRvIII/JAK2/STAT and FAK (Tyr-925) signaling pathways. Afatinib by inhibiting EGFRvIII-cMET cross-activation decreases CSC stemness possibly by downregulating stemness transcription factors Oct3/4 and Nanog. Dotted line indicates possible effects

Using immunofluorescence, we also analyzed the expression of pEGFR (Tyr-1068), pcMET (Tyr-1234/1235), Oct3/4 and Nanog in EGFRvIII tumor xenografts. We observed low expression of these markers in afatinib-treated animal tissues compared to a very high expression in control animal tumors (Fig. 5 D-E). Although TMZ decreased tumor growth, it enriched the expression of pcMET and Nanog (stem cell markers) (Fig. 5 d, e). Overall, the in vivo studies corroborate in vitro observations and reinforce the importance of EGFRvIII/cMET activation in GBM tumorigenesis (Fig. 6).

## Discussion

Despite aggressive therapeutic interventions including surgery, RT and TMZ, the survival of GBM patients has not improved substantially [3]. Most GBM patients develop recurrence that is associated with universal fatality, suggesting an urgent need to understand the disease and develop novel therapies for improving patient survival.

Recent high-throughput data analyses have revealed genetic, epigenetic and mutational features of GBM that have paved the way for personalized medicine. Many studies have established that EGFR and mutant EGFRvIII are amplified/ overexpressed in the majority of GBM patients and are thus important therapeutic targets [6–13]. While strategies that successfully targeted EGFR in other cancers have been tested in GBM, these clinical trials were largely disappointing, possibly due to the compensatory activation of other EGFR family members and RTKs [25]. Phosphatase and tensin homolog (PTEN), a negative regulator of PI3K signaling has also been shown to impact EGFR-targeted therapy outcomes [25]. GBM SP/CSCs show resistance to anti-EGFR therapies by compensatory upregulation of HER2 and HER3. Interestingly, the addition of lapatinib (which inhibits both EGFR and HER2) decreased the SP/CSCs [24]. Therefore, novel therapies targeting concurrent signaling pathways are needed for improved GBM patient outcomes. Aligned with this concept, recently a combination of erlotinib and HGF scavenging antibody L2G7 was shown to significantly decrease tumor growth and improve the OS in EGFRvIII/cMET^+^/HGF^+^/PTEN^−^/^−^ glioma models compared to single agents [64]. Herein, we investigated the efficacy of afatinib and TMZ combination in GBM models overexpressing EGFRvIII in vitro and in vivo. Our studies revealed that together these drugs inhibited proliferation, anchorage-dependent and -independent growth by inducing G_2_/M arrest and senescence of EGFRvIII-expressing GBM cells in vitro. In addition, afatinib with TMZ synergistically inhibited invasion and motility, possibly through inhibiting JAK2/STAT3 and FAK signaling in EGFRvIII-expressing GBM cells. This combination also inhibited cross-activation of cMET and reduced GBM SP/CSCs. Remarkably, our study showed that afatinib and TMZ together not only decreased the tumor burden but also inhibited tumor growth in orthotopic intracranial models.

GBM is highly aggressive and frequently infiltrates/invades surrounding normal brain tissues, hampering complete surgical resection [58] and resulting in progression. It has been previously demonstrated that EGFRvIII signaling can activate JAK2/STAT3 signaling, and inhibiting JAK2 with either a JAK2 inhibitor or siRNA decreased the invasive nature of EGFRvIII cells both in vitro and in vivo [59]. Harada et al. showed that JAK2 signaling plays an important role in developing acquired resistance to erlotinib in lung cancer cells with EGFR-activating mutations [65]. Our study revealed afatinib completely inhibited EGFRvIII-mediated JAK2 signaling. Importantly, afatinib demonstrated prolonged inhibition of EGFRvIII signaling compared to erlotinib, suggesting superior efficacy of afatinib in preventing JAK2-mediated tumorigenic signaling. In addition to JAK2 activation, EGFR-mediated FAK phosphorylation (Y925) and activation also promote migration and invasion of cancer cells [61–63]. Consistent with these results, our study showed U87EGFRvIII cells have elevated pFAK (Y925) levels and greater invasion potential than U87 cells. Remarkably, afatinib treatment completely abrogated EGFRvIII-mediated FAK (Y925) activation/phosphorylation, GBM cell migration, and invasion.

Cancer stem cells, through intrinsic and acquired resistance to CRT, are involved in tumor progression [20, 21]. Many signaling pathways including EGFR are involved in the maintenance of CSCs. Recently, EGFRvIII was shown to cross-activate cMET RTKs [25–27] and enrich GBM CSCs [28, 29]. Using the breast cancer cell line MDA-MB-453 and GBM cell line U-373, treatment with cMET receptor ligand hepatocyte growth factor/scatter factor was shown to impart resistance to doxorubicin/adriamycin and cisplatin [66, 67]. Furthermore, inhibition of cMET by siRNA or the pharmacological inhibitor SU11274 decreased tumorsphere formation in several GBM CSCs in vitro [28]*.* In addition, liposome-conjugated cMET siRNA also decreased GBM tumor growth in an orthotopic mouse model [28]. In concordance with these and our previous results in head and neck squamous cell carcinoma [57], we observed a significant reduction of CSCs with afatinib. Here we conclusively established that afatinib decreases CSCs by abolishing EGFRvIII-cMET signaling.

A recent study showed that the combination of the cMET inhibitor crizotinib with erlotinib significantly decreased stem cell marker expression, neurosphere growth and in vivo tumor growth of human GBM xenografts [68]. While this combination decreased growth in subcutaneous xenograft tumors, the non-permeability of crizotinib through the BBB limited the efficacy in both preclinical and clinical models of brain tumors [68, 69]. Studies have shown that the BBB restricts the availability of not only crizotinib but also most chemotherapeutic drugs to brain tumors and limits their therapeutic efficacy. However, a recent prospective multicenter study of patients with NSCLC and leptomeningeal carcinomatosis showed significant benefits of afatinib, even though only 2.45 ± 2.91% of afatinib penetrated to CSF from blood [70]. Our studies showed afatinib alone has no effects on tumor growth and survival in U87EGFRvIII orthograft-bearing mice. This reduced efficacy may be due to the low dose of afatinib used in our study as opposed to the higher doses used in an NSCLC brain metastases model, which led to tumor regression [71]. Although TMZ reduced growth and overall tumor burden in this model, 60% (4/7) of the animals experienced tumor re-growth, suggesting its limitations as a monotherapy. In contrast, afatinib and TMZ together significantly reduced tumor growth and completely prevented the development of tumor re-growth (5/5). Several studies have shown that chemotherapeutic drugs kill the bulk of differentiating tumor cells, but enrich SP/CSCs, resulting in tumor re-growth. Our results align with these reports as EGFRvIII tumor xenografts showed significant upregulation of CSC markers upon TMZ treatment, but significant downregulation of these markers in mice treated with combined afatinib and TMZ (Fig. 6).

## Conclusion

In summary, our studies demonstrated that the addition of afatinib to TMZ significantly reduced proliferation, clonogenic survival and invasion of U87EGFRvIII GBM cells in vitro and significantly inhibited tumor growth in pre-clinical orthotopic models. Though afatinib was disappointing as a monotherapy in a clinical trial of unselected recurrent GBM patients, it significantly reduced tumor burden when combined with TMZ in U87EGFRvIII xenografts in our pre-clinical mouse model. This work warrants further evaluation of this treatment combination in GBM patients with EGFR amplification or mutant EGFRvIII expression.

## Additional files

Additional file 1: **Figure S1.** TMZ and afatinib synergistically inhibit U87EGFRvIII proliferation (**A-D**). U87MG (3 × 10^3^ cells/well) and U87EGFRvIII (2 × 10^3^ cells/well) were seeded in a 96-well plate and treated with different concentrations of TMZ and afatinib for 48–72 h; viable cells were measured by MTT assay. (**E-F**) Combination treatment significantly decreased the proliferation rate of U87EGFRvIII cells. U87MG and U87EGFRvIII cells were treated with TMZ (25 μM), afatinib (1 μM) or combination for 48 h, and viable cells were measured by MTT assay. Combination index (CI) was calculated using CompuSyn software. (**E**) CI values for non-constant combination: T + A. (**F**) Logarithmic CI graph shows that additional and near synergistic effects of TMZ and afatinib in U87EGFRvIII cells. T - TMZ; A - afatinib. (JPG 215 kb)

Additional file 2: **Figure S2.** Combination of afatinib and TMZ treatment decreases the proliferation of U87EGFRvIII cells by inducing cellular senescence. (**A**) Representative image shows SA-β-galactosidase-positive staining in drug-treated EGFRvIII cells. (**B**) The bar graph shows the mean (±SD) number of senescent cells (*^$^ *P* ≤ 0.05); * significant compared to control; **$** significant compared to TMZ. (**C**) U87EGFRvIII luciferase cells were injected intracranially and treated with afatinib 10 mg/kg/BW (5 days a week p.o.); tumor growth was measured by IVIS imaging at indicated time points. (JPG 139 kb)

Additional file 3: **Figure S3.** U251EGFRvIII brings FAK (Y925) activation. U251EGFRvIII (Tet-inducible system) cells were cultured in the presence and absence of tetracycline and lysates were analyzed for pFAK (Tyr-925), pFAK (Tyr-576/577) and pFAK (Tyr-397). (JPG 44 kb)

Additional file 4: **Table S1.** Antibodies list (DOCX 12 kb)

## Data Availability

Not applicable.

## Abbreviations

BBB: Blood brain barrier
bFGF: Basic fibroblast growth factor
CI: Combination index
CRT: Chemoradiotherapy
CSCs: Cancer stem cells
EGF: Epidermal growth factor
EGFR: Epidermal growth factor receptor
FAK: Focal adhesion kinase
GBM: Glioblastoma
LIF: Leukemia inhibitory factor
NSCLC: Non-small-cell lung cancer
OS: Overall survival
PFS: Progression-free survival
PTEN: Phosphatase and tensin homolog
RT: Radiation therapy
RTKs: Receptor tyrosine kinases
SA-β-gal: Senescence-associated β-galactosidase
SP: Side population
TMZ: Temozolomide
