# Supplementary figures and images for: Afatinib and Temozolomide combination inhibits tumorigenesis by targeting EGFRvIII-cMet signaling in glioblastoma cells
Source: J Exp Clin Cancer Res. 2019 Jun 18;38:266. doi: 10.1186/s13046-019-1264-2 (PMC6582495; doi:10.1186/s13046-019-1264-2)

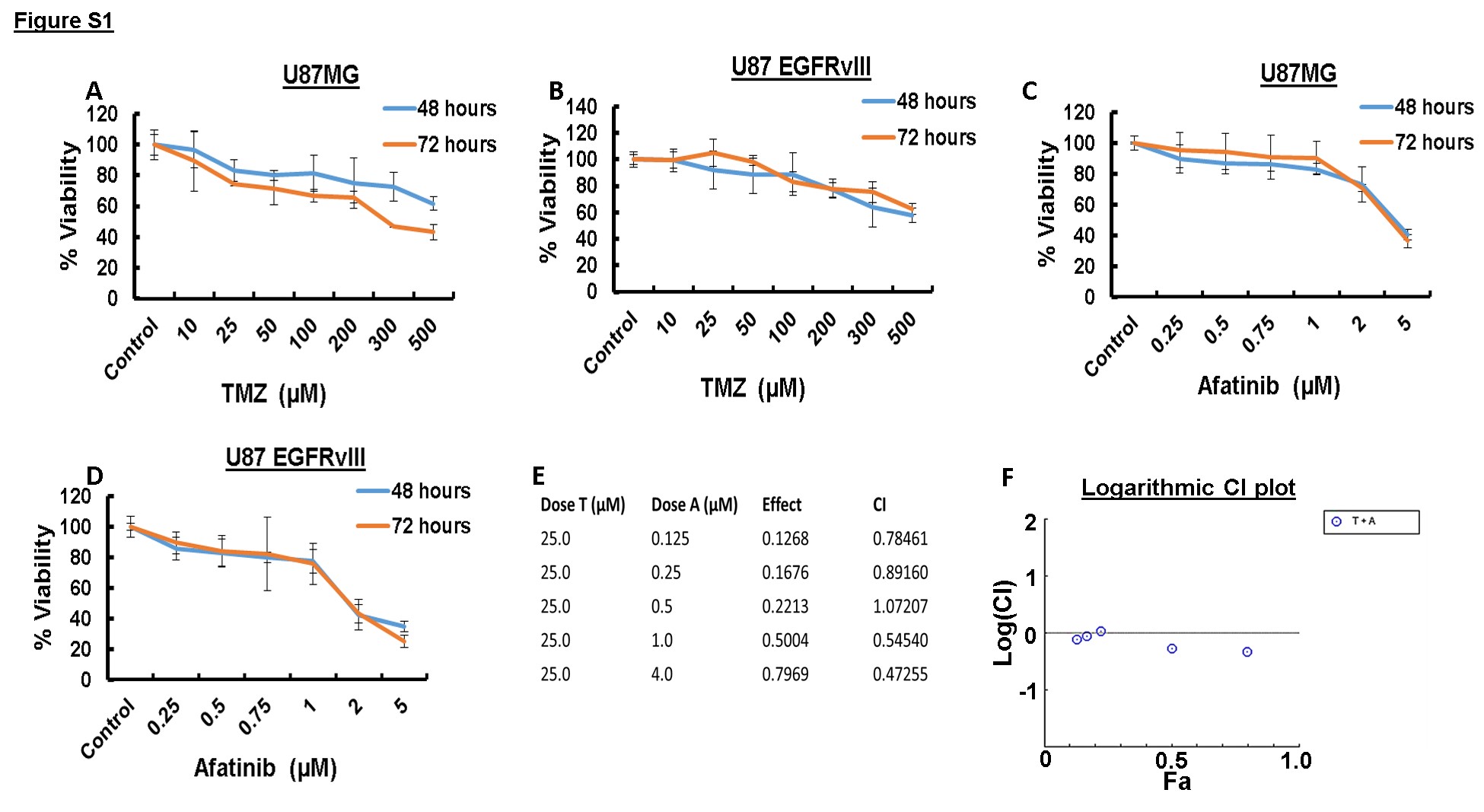

Supplement: Supplementary file 1 — Figure S1. TMZ and afatinib synergistically inhibit U87EGFRvIII proliferation (A-D). U87MG (3 × 10^3^ cells/well) and U87EGFRvIII (2 × 10^3^ cells/well) were seeded in a 96-well plate and treated with different concentrations of TMZ and afatinib for 48–72 h; viable cells were measured by MTT assay. (E-F) Combination treatment significantly decreased the proliferation rate of U87EGFRvIII cells. U87MG and U87EGFRvIII cells were treated with TMZ (25 μM), afatinib (1 μM) or combination for 48 h, and viable cells were measured by MTT assay. Combination index (CI) was calculated using CompuSyn software. (E) CI values for non-constant combination: T + A. (F) Logarithmic CI graph shows that additional and near synergistic effects of TMZ and afatinib in U87EGFRvIII cells. T - TMZ; A - afatinib. (JPG 215 kb) [file 13046_2019_1264_MOESM1_ESM.jpg]

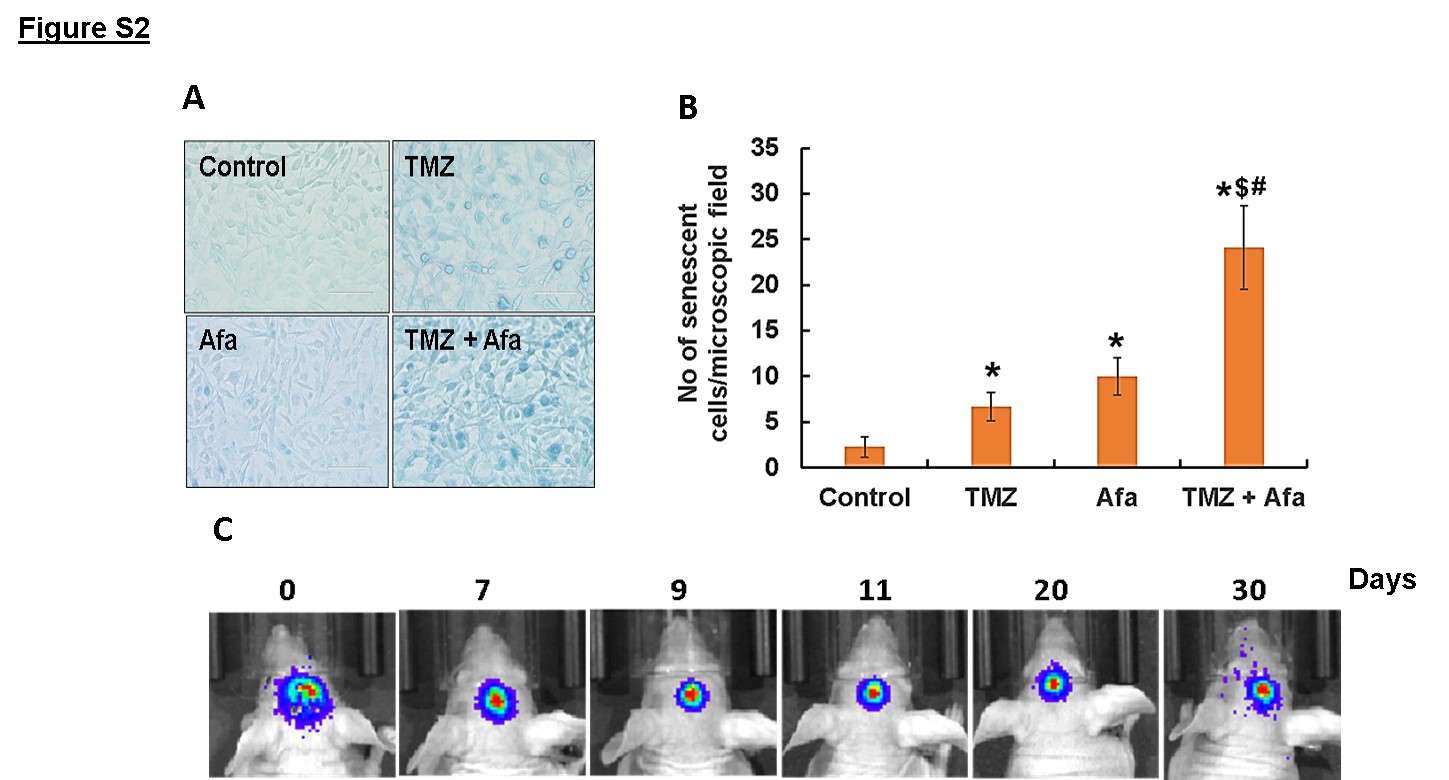

Supplement: Supplementary file 2 — Figure S2. Combination of afatinib and TMZ treatment decreases the proliferation of U87EGFRvIII cells by inducing cellular senescence. (A) Representative image shows SA-β-galactosidase-positive staining in drug-treated EGFRvIII cells. (B) The bar graph shows the mean (±SD) number of senescent cells (*$ P ≤ 0.05); * significant compared to control; $ significant compared to TMZ. (C) U87EGFRvIII luciferase cells were injected intracranially and treated with afatinib 10 mg/kg/BW (5 days a week p.o.); tumor growth was measured by IVIS imaging at indicated time points. (JPG 139 kb) [file 13046_2019_1264_MOESM2_ESM.jpg]

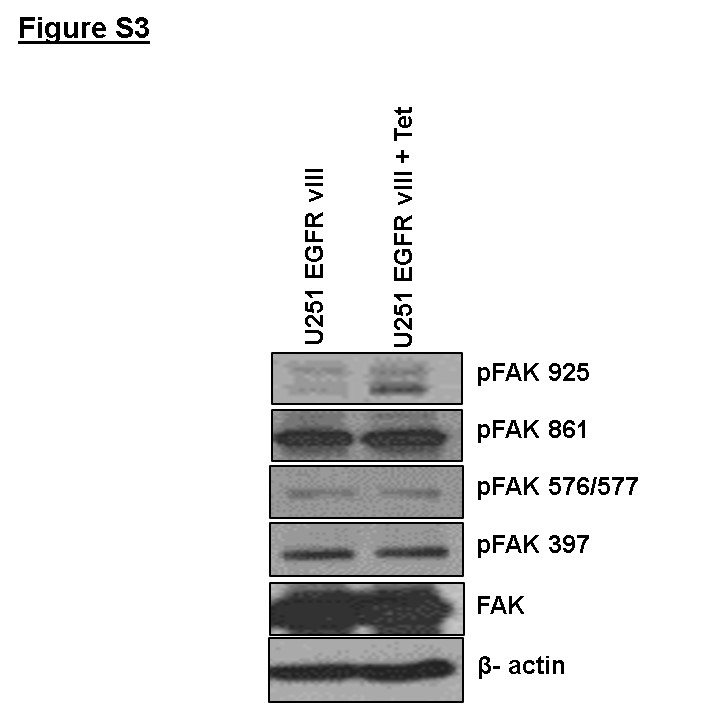

Supplement: Supplementary file 3 — Figure S3. U251EGFRvIII brings FAK (Y925) activation. U251EGFRvIII (Tet-inducible system) cells were cultured in the presence and absence of tetracycline and lysates were analyzed for pFAK (Tyr-925), pFAK (Tyr-576/577) and pFAK (Tyr-397). (JPG 44 kb) [file 13046_2019_1264_MOESM3_ESM.jpg]
