# Supplementary material for: Afatinib and Temozolomide combination inhibits tumorigenesis by targeting EGFRvIII-cMet signaling in glioblastoma cells
Source: J Exp Clin Cancer Res. 2019 Jun 18;38:266. doi: 10.1186/s13046-019-1264-2 (PMC6582495; doi:10.1186/s13046-019-1264-2)
Supplement: Supplementary file 4 — Table S1. Antibodies list (DOCX 12 kb) [file 13046_2019_1264_MOESM4_ESM.docx]

**Table S1: Antibodies list**

| **S.no** | **Antibody name** | **Manufacturer** | **Cat.no** |
| --- | --- | --- | --- |
| 1 | EGF receptor | CST | 4267 |
| 2 | EGF receptor vIII | CST | 64952 |
| 3 | P-EGF receptor (Y1068) | CST | 3777 |
| 4 | FAK | Santa cruz | SC-558 |
| 5 | pFAK (Y397) | CST | 8556 |
| 6 | pFAK (Y576/577) | CST | 3281 |
| 7 | pFAK (Y925) | CST | 3284 |
| 8 | pJAK2 (Y1007/1008) | CST | 3771 |
| 9 | cMET | CST | 8198 |
| 10 | pcMET (Y1234/1235) | CST | 3077 |
| 11 | AKT | CST | 4691 |
| 12 | pAKT (S473) | CST | 4060 |
| 13 | STAT3 | CST | 12640 |
| 14 | pSTAT3 | CST | 9145 |
| 15 | Nestin | Abcam | Ab22035 |
| 16 | SOX9 | CST | 82630 |
| 17 | CD15/SSEA-1 | Invitrogen | 41-1200 |
| 18 | Oct3/4 | Santa cruz | SC-5279 |
| 19 | Nanog | Santa cruz | SC-30329 |
| 20 | β-actin | Sigma | A1978 |
